# Supplementary material for: Prognosis of Pneumonia in Head and Neck Squamous Cell Carcinoma Patients Who Received Concurrent Chemoradiotherapy
Source: Biomedicines. 2024 Jul 4;12(7):1480. doi: 10.3390/biomedicines12071480 (PMC11274903; doi:10.3390/biomedicines12071480)
Supplement: Supplementary file 1 [file biomedicines-12-01480-s001.zip › biomedicines-3070311-supplementary.pdf]

Supplementary Materials

Table S1. Diagnosis codes of cancer.

Cancer	ICD-9	ICD-10	Diagnosis
**Hypopharynx**	1480	C130	Malignant neoplasm of postcricoid region
	1481	C12	Malignant neoplasm of pyriform sinus
	1482	C131	Malignant neoplasm of aryepiglottic fold, hypopharyngeal aspect
	1483	C132	Malignant neoplasm of posterior wall of hypopharynx
	1488	C138	Malignant neoplasm of overlapping sites of hypopharynx
	1489	C139	Malignant neoplasm of hypopharynx, unspecified
**Larynx**	1610	C320	Malignant neoplasm of glottis
	1611	C321	Malignant neoplasm of supraglottis
	1612	C322	Malignant neoplasm of subglottis
	1613	C323	Malignant neoplasm of laryngeal cartilage
	1618	C328	Malignant neoplasm of overlapping sites of larynx
	1619	C329	Malignant neoplasm of larynx, unspecified
**Nasopharynx**	1470	C110	Malignant neoplasm of superior wall of nasopharynx
	1471	C111	Malignant neoplasm of posterior wall of nasopharynx

	1472	C112	Malignant neoplasm of lateral wall of nasopharynx
	1473	C113	Malignant neoplasm of anterior wall of nasopharynx
	1478	C118	Malignant neoplasm of overlapping sites of nasopharynx
	1479	C119	Malignant neoplasm of nasopharynx, unspecified
**Oral Cavity**	1400	C000	Malignant neoplasm of external upper lip
	1401	C001	Malignant neoplasm of external lower lip
	1403	C003	Malignant neoplasm of upper lip, inner aspect
	1404	C004	Malignant neoplasm of lower lip, inner aspect
	1405	C005	Malignant neoplasm of lip, unspecified, inner aspect
	1406	C006	Malignant neoplasm of commissure of lip, unspecifie
	1408	C008	Malignant neoplasm of overlapping sites of lip
	1409	C002	Malignant neoplasm of external lip, unspecified
	1409	C009	Malignant neoplasm of lip, unspecified
	1411	C020	Malignant neoplasm of dorsal surface of tongue
	1412	C021	Malignant neoplasm of border of tongue
	1413	C022	Malignant neoplasm of ventral surface of tongue
	1414	C023	Malignant neoplasm of anterior two-thirds of tongue, part unspecified

1415	C028	Malignant neoplasm of overlapping sites of tongue
1418	C028	Malignant neoplasm of overlapping sites of tongue
1419	C029	Malignant neoplasm of tongue, unspecified
1430	C030	Malignant neoplasm of upper gum
1431	C031	Malignant neoplasm of lower gum
1438	C039	Malignant neoplasm of gum, unspecified
1439	C039	Malignant neoplasm of gum, unspecified
1440	C040	Malignant neoplasm of anterior floor of mouth
1441	C041	Malignant neoplasm of lateral floor of mouth
1448	C048	Malignant neoplasm of overlapping sites of floor of mouth
1449	C049	Malignant neoplasm of floor of mouth, unspecified
1450	C060	Malignant neoplasm of cheek mucosa
1451	C061	Malignant neoplasm of vestibule of mouth
1452	C050	Malignant neoplasm of hard palate
1456	C062	Malignant neoplasm of retromolar area
1458	C0680	Malignant neoplasm of overlapping sites of unspecified parts of mouth

	1458	C0689	Malignant neoplasm of overlapping sites of other parts of mouth
	1459	C069	Malignant neoplasm of mouth, unspecified
**Oropharynx**	1410	C01	Malignant neoplasm of base of tongue
	1416	C024	Malignant neoplasm of lingual tonsil
	1453	C051	Malignant neoplasm of soft palate
	1454	C052	Malignant neoplasm of uvula
	1460	C098	Malignant neoplasm of overlapping sites of tonsil
	1460	C099	Malignant neoplasm of tonsil, unspecified
	1461	C090	Malignant neoplasm of tonsillar fossa
	1462	C091	Malignant neoplasm of tonsillar pillar (anterior) (posterior)
	1463	C100	Malignant neoplasm of vallecula
	1464	C101	Malignant neoplasm of anterior surface of epiglottis
	1465	C108	Malignant neoplasm of overlapping sites of oropharynx
	1466	C102	Malignant neoplasm of lateral wall of oropharynx
	1467	C103	Malignant neoplasm of posterior wall of oropharynx
	1468	C104	Malignant neoplasm of branchial cleft

	1468	C108	Malignant neoplasm of overlapping sites of oropharynx
	1469	C109	Malignant neoplasm of oropharynx, unspecified
**Esophageal cancer**	1500		Malignant neoplasm of cervical esophagus
	1503	C153	Malignant neoplasm of upper third of esophagus
	1501		Malignant neoplasm of thoracic esophagus
	1504	C154	Malignant neoplasm of middle third of esophagus
	1502		Malignant neoplasm of abdominal esophagus
	1505	C155	Malignant neoplasm of lower third of esophagus
	1508	C158	Malignant neoplasm of other specified part of esophagus
	1509	C159	Malignant neoplasm of esophagus, unspecified
**Salivary gland cancer**	1420	C07	Malignant neoplasm of parotid gland
	1421	C080	Malignant neoplasm of submandibular gland
	1422	C081	Malignant neoplasm of sublingual gland
	1428		Malignant neoplasm of other major salivary glands
	1429	C089	Malignant neoplasm of salivary gland, unspecified

Table S2. Diagnosis codes of pneumonia.

ICD-9	ICD-10	Diagnosis
**481**	**J13**	Pneumonia due to Streptococcus pneumoniae
**481**	**J181**	Lobar pneumonia, unspecified organism
**4820**	**J150**	Pneumonia due to Klebsiella pneumoniae
**4821**	**J151**	Pneumonia due to Pseudomonas
**4822**	**J14**	Pneumonia due to Hemophilus influenzae
**48230**	**J154**	Pneumonia due to other streptococci
**48231**	**J154**	Pneumonia due to other streptococci
**48232**	**J153**	Pneumonia due to streptococcus, group B
**48239**	**J154**	Pneumonia due to other streptococci
**48240**	**J1520**	Pneumonia due to staphylococcus, unspecified
**48241**	**J15211**	Pneumonia due to Methicillin susceptible Staphylococcus aureus
**48249**	**J1529**	Pneumonia due to other staphylococcus
**48281**	**J158**	Pneumonia due to other specified bacteria
**48282**	**J155**	Pneumonia due to Escherichia coli
**48283**	**J156**	Pneumonia due to other aerobic Gram-negative bacteria
**48289**	**J158**	Pneumonia due to other specified bacteria
**4829**	**J159**	Unspecified bacterial pneumonia

**4848**	**J17**	Pneumonia in diseases classified elsewhere
**485**	**J180**	Bronchopneumonia, unspecified organism
**486**	**J188**	Other pneumonia, unspecified organism
**486**	**J189**	Pneumonia, unspecified organism
**507**	**J69**	*Pneumonitis due to solids and liquids
**5070**	**J690**	Pneumonitis due to inhalation of food and vomit
**5071**	**J691**	Pneumonitis due to inhalation of oils and essences
**5078**	**J698**	Pneumonitis due to inhalation of other solids and liquids
	**J9589**	Other postprocedural complications and disorders of respiratory system, not elsewhere classified
